# Snail promotes resistance to enzalutamide through regulation of androgen receptor activity in prostate cancer

**DOI:** 10.18632/oncotarget.10476

**Published:** 2016-07-07

**Authors:** Kathryn E. Ware, Jason A. Somarelli, Daneen Schaeffer, Jing Li, Tian Zhang, Sally Park, Steven R. Patierno, Jennifer Freedman, Wen-Chi Foo, Mariano A. Garcia, Andrew J. Armstrong

**Affiliations:** ^1^ Department of Medicine, Division of Medical Oncology, Duke University Medical Center, Durham, NC, USA; ^2^ Department of Genitourinary Oncology, Duke Cancer Institute, Duke University Medical Center, Durham, NC, USA; ^3^ Department of Oncology, Translational Research, Janssen Research and Development, Spring House, PA, USA; ^4^ Department of Hematology and Oncology, Wake Forest School of Medicine, Winston-Salem, NC, USA; ^5^ Department of Molecular Genetics and Microbiology, Duke University Medical Center, Durham, NC, USA; ^6^ Department of Biochemistry and Molecular Biology, The University of Texas Medical Branch, Galveston, TX, USA; ^7^ Department of Pharmacology and Cancer Biology, Duke University, Durham, NC, USA; ^8^ Department of Pathology, Duke University, Durham, NC, USA

**Keywords:** castration resistance, metastasis, enzalutamide, androgen receptor, Snail

## Abstract

Treatment with androgen-targeted therapies can induce upregulation of epithelial plasticity pathways. Epithelial plasticity is known to be important for metastatic dissemination and therapeutic resistance. The goal of this study is to elucidate the functional consequence of induced epithelial plasticity on AR regulation during disease progression to identify factors important for treatment-resistant and metastatic prostate cancer. We pinpoint the epithelial plasticity transcription factor, Snail, at the nexus of enzalutamide resistance and prostate cancer metastasis both in preclinical models of prostate cancer and in patients. In patients, Snail expression is associated with Gleason 9-10 high-risk disease and is strongly overexpressed in metastases as compared to localized prostate cancer. Snail expression is also elevated in enzalutamide-resistant prostate cancer cells compared to enzalutamide-sensitive cells, and downregulation of Snail re-sensitizes enzalutamide-resistant cells to enzalutamide. While activation of Snail increases migration and invasion, it is also capable of promoting enzalutamide resistance in enzalutamide-sensitive cells. This Snail-mediated enzalutamide resistance is a consequence of increased full-length AR and AR-V7 expression and nuclear localization. Downregulation of either full-length AR or AR-V7 re-sensitizes cells to enzalutamide in the presence of Snail, thus connecting Snail-induced enzalutamide resistance directly to AR biology. Finally, we demonstrate that Snail is capable of mediating-resistance through AR even in the absence of AR-V7. These findings imply that increased Snail expression during progression to metastatic disease may prime cells for resistance to AR-targeted therapies by promoting AR activity in prostate cancer.

## INTRODUCTION

Prostate cancer is responsible for over 80 deaths per day in the US [1]. Androgen receptor (AR) is involved in normal prostate development, prostate cancer progression, and response to hormonal-based therapies, such as enzalutamide. Castration resistant prostate cancer (CRPC) is defined as progressive disease, often based on biochemical (serum PSA rises) or radiographic evidence of metastatic spread, despite castrate levels of testosterone [2]. During CRPC progression and resistance to hormonal-targeted therapies, continued dependence on the AR is a common occurrence, and sustained activation of the AR transcriptional program is observed [3, 4]. Furthermore, the evolution of CRPC is frequently accompanied by emergence of AR variants that lack the ligand-binding domain. These AR variants result from deletions in the *AR* gene or alternative splicing of AR pre-mRNA [5–8]. The best-characterized, clinically relevant AR variant is AR-V7 [9–17]. Notably, the expression of AR-V7 is sufficient to confer resistance to androgen deprivation therapy (ADT) in preclinical models [10–13, 18, 19]. Most importantly, detection of AR-V7 in circulating tumor cells (CTCs) from men with CRPC was strongly associated with a lack of response or clinical benefit to either enzalutamide or abiraterone acetate, indicating that clinical resistance to these agents is at least associated with AR-V7 expression [19, 20]. However, it is important to note that AR-V7 is typically expressed as the minor AR isoform in comparison to full-length AR (AR-FL), and restoration of AR signaling and recurrence can occur in the absence of AR-V7 [19].

Preclinical models have shown that AR-FL and AR-V7 overexpression is associated with induction of a mesenchymal or stem-like phenotype known to promote metastasis [21–24], and death from prostate cancer is almost uniformly due to complications associated with metastasis. Therefore, understanding this metastatic propensity is of paramount clinical importance and critical to developing new therapies. Prior work has identified higher levels of mesenchymal biomarkers in metastatic prostate cancer [25–28]. Cells characterized by mesenchymal biomarkers are associated with resistance to radiation, chemotherapeutics and AR-targeted therapies that are commonly used to treat prostate cancer [29–32]. It is important to note that most transformed cells exist in phenotypic states in a continuous spectrum of epithelial and mesenchymal properties and often are capable of shifting within the spectrum based on selective pressures. Notably, prostate cancer treatment itself can induce this epithelial plasticity toward a less epithelial phenotype, and loss of epithelial differentiation markers is associated with aggressive disease and treatment resistance in patients [30, 32]. Thus, there is evidence that both AR and drivers of epithelial plasticity can be rapidly upregulated upon AR-targeted treatment [30, 33]. However, little is known about how epithelial plasticity programs impact AR activity during disease progression. In the present study, we define a novel functional role for master epithelial plasticity driver, Snail, in AR regulation during prostate cancer progression to treatment resistant disease. We demonstrate that Snail mRNA and protein is associated with aggressive, metastatic prostate cancer in patients. Additionally, in a cell line-based model of enzalutamide resistance, Snail expression is upregulated, along with AR-FL and AR-V7, and Snail depletion restores enzalutamide sensitivity. Likewise, ectopic expression of Snail promotes both AR expression and nuclear localization. Consequently, Snail activation leads to enzalutamide resistance by regulating AR activity, as RNAi-mediated silencing of either AR-FL or AR-V7 blocked Snail-mediated enzalutamide resistance. Importantly, Snail is capable of mediating resistance through AR even in the absence of AR-V7. Together these results mechanistically identify a novel role for Snail in mediating enzalutamide resistance through the regulation of AR expression and localization.

## RESULTS

### Prostate cancer cells resistant to enzalutamide exhibit higher Snail expression

**Figure 1:**
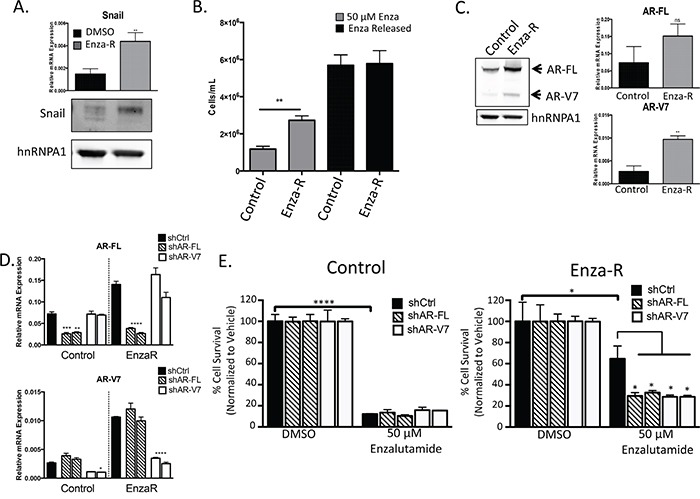
Enzalutamide resistance is mediated through increased expression of AR and AR-V7 **A.** Snail mRNA and protein expression measured by qPCR and western blot, respectively, ** p<0.01; student's t-test. **B.** LNCaP95 control or EnzaR cells were cultured in RPMI 10% CS-FBS containing DMSO or 50 μM enzalutamide, ** = p<0.005; student's t-test. Mean cell counts are plotted along with standard error of the mean (SEM). **C.** Whole cell lysates were collected and immunoblotted for AR-FL, AR-V7 and hnRNPA1 as a loading control. Relative mRNA levels of AR-FL and AR-V7 were measured using qPCR and normalized to GAPDH, ** = p<0.005; student's t-test. **D.** Relative mRNA knock down of AR-FL or AR-V7 with two independent shRNA constructs, * = p<0.05, **** = p< 0.0001; one-way ANOVA. **E.** LNCaP95 control or EnzaR cells with AR knock down were cultured in media containing DMSO or 50 μM enzalutamide and cells were enumerated as above, * = p<0.05, **** = p<0.0001; one-way ANOVA.

Enzalutamide is a potent inhibitor of AR signaling that is currently used as a first line therapy to treat prostate cancer patients with metastatic CRPC. Although most patients initially respond to enzalutamide treatment, all eventually progress within several years [34], and some men develop rapid progression and resistance within several months. We established an *in vitro* model of enzalutamide resistance using LNCaP95 cells. These cells were originally derived from the LNCaP model through passaging in low androgen conditions [7, 35, 36], and have an intact AR genome and lower levels of AR-V7 as compared to full length AR. This cell line models the human condition of patients who have AR-V7 expression, which is enriched in patients following castrate conditions and typically is expressed in a low ratio compared to full length AR [19]. We further serially passaged this cell line under increasing concentrations of enzalutamide up to 50 μM enzalutamide, which is two-fold higher than the clinically relevant dose. LNCaP95 enzalutamide-resistant (Enza-R) cells exhibited increased Snail expression compared to control cells (Figure 1A). Snail expression was not impacted by acute treatment with a clinically relevant dose of enzalutamide, suggesting that Snail is acquired through chronic treatment with enzalutamide (Supplementary Figure S1A). EnzaR cells were less rounded, more spindle-like in morphology and displayed a more scattered phenotype (Supplementary Figure S1B). In addition to increases in Snail expression, we also detected more nuclear Snail in Enza-R cells compared to control cells (Supplementary Figure S1C). Due to these changes in Snail expression and localization and cell morphology, we determined whether these cells had undergone a partial epithelial-to-mesenchymal transition (EMT). To assay for EMT-like changes we chose to measure by qPCR a panel of genes associated with either an epithelial or mesenchymal cell state. Although Snail is known to regulate genes involved in EMT [37, 38], we did not observe a change in these traditional EMT-associated Snail targets, such as loss of E-cadherin or gain of N-cadherin, vimentin, or other regulators of EMT in Enza-R cells (Supplementary Figure S1D).

### Snail confers resistance to enzalutamide through upregulation of both AR-FL and AR-V7

LNCaP95 Enza-R cells have a significantly higher proliferation rate over DMSO-treated, passage control cells when treated with enzalutamide (Figure 1B). Interestingly, Enza-R cells released from enzalutamide treatment have a further increase in proliferation, despite being adapted to chronic culture with enzalutamide (Figure 1B). Therefore, Enza-R cells remain responsive to AR full-length (FL) blockade, which suggests that enzalutamide remains an antagonist and that AR-FL continues to be important for cell growth in Enza-R cells. Likewise, we verified that Enza-R cells did not acquire the agonistic F876L mutation during progression to enzalutamide resistance (Supplementary Figure S2). We next examined expression levels of AR-FL and AR-V7, since AR signaling is known to confer resistance to ADT through AR amplification or upregulation of AR-V7. In Enza-R cells, both AR-FL and AR-V7 were upregulated compared to control cells (Figure 1C), further suggesting that AR is involved in enzalutamide resistance.

Next, we examined whether knocking down AR-FL or AR-V7 impacted the growth of control and Enza-R cells when treated with enzalutamide. We were able to specifically target AR-FL or AR-V7 each with two independent shRNAs (Figure 1D, Supplementary Figure S2B). LNCaP95 Enza-R cells expressing shRNA targeting AR-FL or AR-V7 invoked a similar decrease in enzalutamide resistance compared to Enza-R cells expressing a control (Ctrl) shRNA (Figure 1E). Interestingly, depletion of AR-FL or AR-V7 did not further increase sensitivity to enzalutamide in LNCaP95 control cells (Figure 1E). These data indicate that enzalutamide resistance remains mediated through an increase in AR expression.

**Figure 2:**
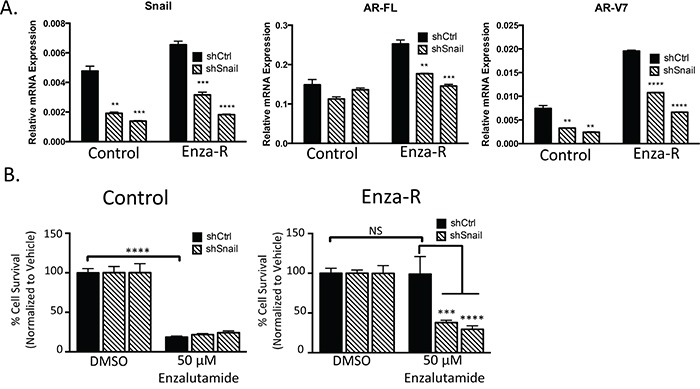
Snail upregulation mediates AR upregulation and enzalutamide resistance **A.** LNCaP95 control or EnzaR cells, stably transduced with control or two independent Snail targeted shRNAs, were analyzed for relative amounts of mRNA for AR-FL, AR-V7 and Snail. Amounts were determined by qPCR and were normalized to GAPDH, * = p<0.05; student's t-test. **B.** LNCaP95 control or EnzaR cells expressing control or 2 independent Snail shRNAs were cultured in media containing DMSO or 50 μM enzalutamide and cells were enumerated, *** = p<0.0005, **** = p<0.0001; one-way ANOVA.

Based on our observation that both Snail and AR are increased in enzalutamide-resistant cells, we sought to assess whether Snail played a role in the induction of AR in Enza-R cells and the impact of Snail knockdown on enzalutamide resistance. Control cells maintained expression of AR-FL in the presence of Snail knockdown and exhibited relatively low levels of AR-V7 that was further reduced with Snail knockdown. Stable knockdown of Snail did not further sensitize control cells to enzalutamide. In contrast, Snail depletion with shRNAs inhibited both AR-FL and AR-V7 expression in EnzaR cells (Figure 2A, Supplementary Figure S2C). Consequently, Snail knockdown led to a significant decrease in enzalutamide resistance (~60%, p≤0.005) in LNCaP95 Enza-R cells (Figure 2B). These data pinpoint Snail as a factor that is upregulated and important for enzalutamide resistance, in part, through the regulation of AR expression. Together these results suggest that increasing total AR levels renders cells resistant to enzalutamide treatment and suggests that Snail is acting upstream of AR and AR variant activity to mediate this acquired enzalutamide resistance.

### Snail activation induces a mesenchymal gene signature and enhances migration and invasion

**Figure 3:**
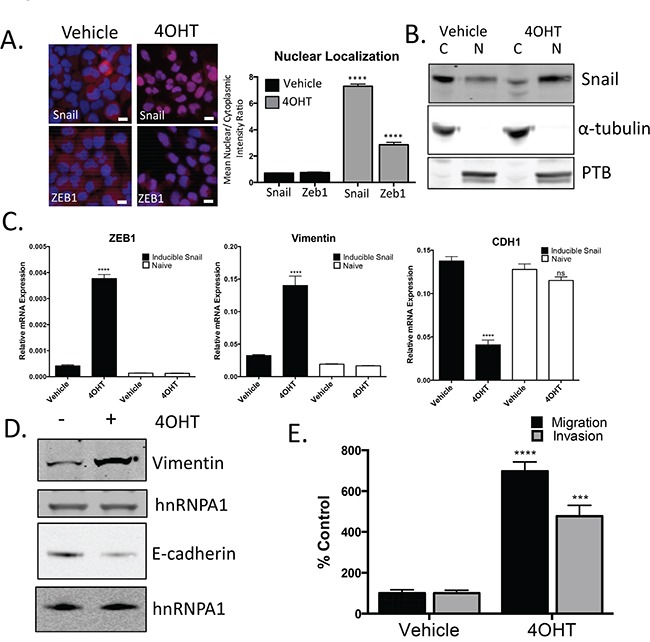
Snail activation increases the migratory and invasive potential of prostate cancer cells **A.** Representative images and quantification for Snail and ZEB1 nuclear localization following treatment with 4OHT (20 nM). Blue: Hoechst stained nuclei; Red: Snail or ZEB1; scale bar = 20 μm. **B.** Analysis of cytoplasmic (C) and nuclear (N) localization of Snail by western blot. Alpha-tubulin and PTB were probed as loading controls and compartment controls for cytoplasmic and nuclear fractionation. **C.** Total RNA was extracted from LNCAP95 naïve and LNCaP95-Snail cells. Relative mRNA levels of Zeb1, vimentin and CDH1 were normalized to GAPDH, **** p<0.0001; one-way ANOVA. **D.** Snail activated cells have increased vimentin and decreased E-cadherin expression by western blot analysis. **E.** Tamoxifen treated, LNCaP95Snail(+) cells are more migratory and invasive. Cells migrating through uncoated (migration) and Matrigel-coated (invasion) Boyden chambers were stained with Phalloidin and cell number was quantified using ImageJ. *** p<0.0005, **** p<0.0001; student's t-test.

Expression of Snail and AR are both upregulated during enzalutamide resistance. To explore the impact of Snail activation on enzalutamide resistance and AR signaling, we developed a tamoxifen-inducible Snail model in enzalutamide sensitive, LNCaP95 prostate cancer cells. The addition of tamoxifen (4OHT) was sufficient to induce nuclear localization of Snail (Figure 3A, 3B, Supplementary Figure S3A). Nuclear Snail was functionally active as shown by induction of *ZEB1* and *VIM* and repression of *CDH1* (Figure 3C, 3D). Importantly, tamoxifen alone did not induce gene changes in non-Snail transduced cells (Figure 3C, open bars). We next wanted to assess the biological impact of these gene changes associated with cancer cell aggressiveness. Therefore, we examined how Snail activation alters cellular migration and invasion. Snail activation significantly increased migration and invasion by 7.0 (p=<0.0001) and 4.8 (p=0.0002) fold, respectively. (Figure 3E). These results confirm that Snail is activated with 4OHT treatment, which increases the metastatic potential of these prostate cancer cells.

### Snail expression correlates with aggressive primary disease and is further enriched in metastatic prostate cancer

**Figure 4:**
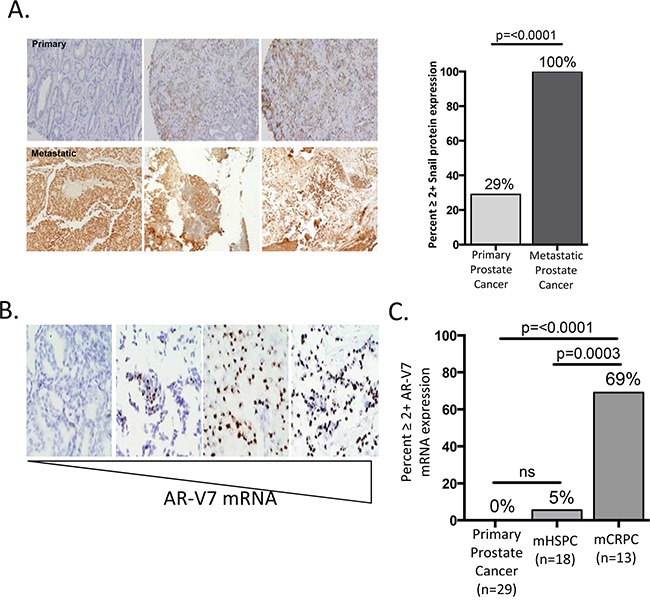
Snail expression is enriched in metastatic biopsies **A.** Snail is strongly expressed in 100% of prostate cancer metastases compared to just 29% of primary tumors. Images are representative of Snail staining in three primary (top) and three metastatic (bottom) prostate cancer tissues. **B.** Representative images of varying levels of AR-V7 mRNA from RNA-ISH staining on metastatic prostate samples. Metastatic images from left to right are from the lymph (score 0), brain (score 3 and 4) and lymph (score 4). **C.** AR-V7 mRNA is expressed in 0% (n=29) of primary tumors, 5% (n=18) of metastatic hormone-sensitive tumors (mHSPC) and 69% (n=10) of metastatic castration resistant tumors (mCRPC).

Our results indicate that Snail over-expression induces a more migratory and invasive phenotype in prostate cancer cells. Based on this, we hypothesized that Snail would be associated with clinically aggressive disease. To determine whether Snail is associated with aggressive prostate cancer in clinical disease, we analyzed RNA-Seq data from The Cancer Genome Atlas (TCGA) and found that Snail RNA levels significantly correlated with increasing Gleason score (Supplementary Figure S4). Snail RNA was particularly high in the most aggressive Gleason 10 tumors, which are highly associated with recurrence after local therapy and with lethal disease [39]. Importantly, expression of other mesenchymal transcription factors, Slug, Zeb1, Zeb2, and Twist, did not correlate with Gleason score (Supplementary Figure S4). Given the positive relationship between higher Gleason score and Snail expression, we next asked whether Snail was upregulated in metastatic disease compared to primary tumors. We stained and compared 31 metastatic biopsies (Supplementary Figure S5) to 188 primary biopsies (previously characterized [40]) for Snail expression by immunohistochemistry from men with prostate cancer. Remarkably, we observed strong nuclear Snail staining (≥2+) in 100% of metastatic biopsies as compared to just 29% in localized prostate cancer (p<0.0001) [40] (Figure 4A). We also examined total AR, cytokeratin and vimentin expression in the 31 metastatic samples by IHC. Interestingly, these samples had strong, but diffuse expression of the epithelial biomarker, cytokeratin (CK), but had low or no expression of the mesenchymal marker, vimentin in tumor cells (Supplementary Figure S5A). These data suggest that the metastases exist in a dual epithelial (CK+, vimentin-) and mesenchymal state (Snail+), similar to the phenotype we have previously demonstrated in CTCs captured from men with metastatic CRPC [31]. High nuclear AR expression was also observed in the majority (94%) of metastatic samples (Supplementary Figure S5A, S6). Interestingly, metastatic samples from men with both hormone-sensitive (n=18) and castration-resistant (n=13) disease exhibited a similar high expression of Snail, suggesting that Snail overexpression is acquired early during metastatic disease progression (Supplementary Figure S5A).

As discussed above, we observed high nuclear staining of both Snail (100%) and total AR (94%) in our metastatic samples (Supplementary Figure S6) and demonstrated Snail contributes to both AR-FL and AR-V7 expression *in vitro*. We next asked whether AR-V7 was expressed in clinical samples of prostate cancer. To assess the levels of AR-V7 expression, we used RNA in-situ hybridization (ISH) to measure AR-V7 mRNA in our metastatic biopsies [19]. Given that low level (1+ AR-V7) expression was detected in AR negative control cell lines, only 2+ or greater AR-V7 expression scores were determined to be positive with this assay. AR-V7 mRNA (2-4+ expression) was detected in 10 of 31 metastatic samples (Figure 4B, Supplementary Figure S5B, S5D). This is in contrast to primary prostate cancer, in which AR-V7 was detected in 0% (n=29) of samples. Thus, AR-V7 expression is significantly (p=<0.0001) enriched in metastatic CRPC samples (Figure 4C, Supplementary Figure S5B, S5C). Of the AR-V7 positive samples, all brain metastases (n=3) and post-abiraterone/enzalutamide CRPC (n=4) metastases were positive for AR-V7, and 9 of 13 (69%) CRPC biopsies were positive for AR-V7 (Supplementary Figure S5D). In contrast to Snail staining, which was expressed in all hormone-sensitive metastatic biopsies, AR-V7 was rarely expressed in these castrate-sensitive metastases, with 1/18 (5%) cases demonstrating only moderate AR-V7 expression (p=0.0003 vs CRPC samples). This suggests AR-V7 expression is selected upon following AR-targeted therapy, and that Snail overexpression in patients coincides with metastatic progression, even before the onset of castration-resistance and AR-V7 detection. Given this timing, we hypothesize Snail functions to promote metastasis and AR-FL expression. However, disease progression following multiple androgen and AR-targeted therapies selects for cells with AR-V7 expression, which permits Snail to contribute to expression of both AR-FL and AR-V7 to promote treatment resistance.

### Snail activation increases AR expression and enzalutamide resistance

**Figure 5:**
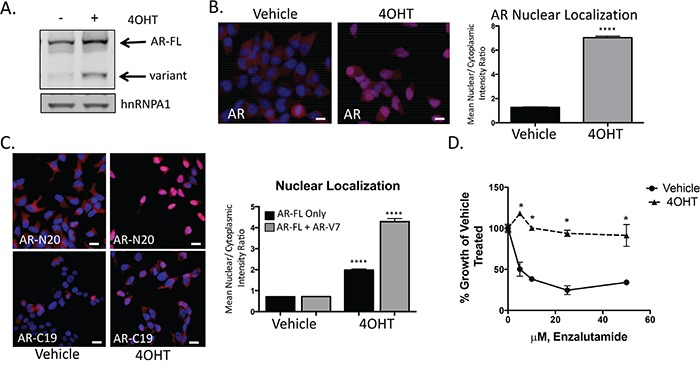
Snail activation increases AR expression and enzalutamide resistance **A.** Tamoxifen (4OHT) treated, LNCaP95Snail(+) cells have increased AR-FL and AR-variant expression by western blot analysis. **B.** Representative images from immunofluorescence staining for total AR (Blue: Hoechst stained nuclei; Red: AR) and quantification of nuclear to cytoplasmic intensity from immunofluorescence staining of AR; scale bar = 20 μm. **C.** Immunofluorescence images from N-terminus (AR-N20) and C-terminus (AR-C19) AR antibodies and quantification of nuclear/cytoplasmic intensity; scale bar = 20 μm. **D.** Tamoxifen treated, LNCaP95Snail(+) cells are more resistance to enzalutamide inhibited growth. Cell growth was quantified using WST-1 after 10 days in the presence of increasing concentrations of enzalutamide. Results are presented as means and SEM of 3 experiments performed in triplicate, * p<0.001; student's t-test.

In our metastatic biopsies, we commonly observed high nuclear expression of Snail and AR, and both Snail and AR were upregulated in our pre-clinical model of enzalutamide resistance. Consequently, we examined AR regulation in the presence of activated Snail in our tamoxifen-induced model. We found that both AR-FL and AR-V7 were upregulated when Snail was activated (Figure 5A). In addition to an increase in expression of AR, we also observed an increase in AR nuclear localization (Figure 5B, Supplementary Figure S3B). To estimate changes in AR-FL vs. AR-V7 localization, we used N-terminal or C-terminal AR antibodies to compare the frequency of total AR to AR-FL nuclear localization, respectively. AR nuclear localization was similar between N-terminal and C-terminal staining in control cells (Figure 5C, Supplementary Figure S3C). Analogous to Figure 5B, cells with activated Snail had increased AR nuclear localization compared to control cells. Nuclear localization was increased using both the N-terminal and C-terminal AR antibodies; however, we detected over 50% more AR nuclear localization with the N-terminal AR antibody than with the C-terminal AR antibody when Snail was activated (Figure 5C, Supplementary Figure S3C). We conclude from this that Snail activation switches the AR nuclear profile from balanced N-terminal and C-terminal AR expression in control cells to predominant N-terminal AR staining in Snail-activated cells, indicating a decrease in the proportion of nuclear AR-FL localization compared to total AR nuclear localization. This change in proportion of AR-FL nuclear localization suggests that more AR-V7 is located in the nucleus upon Snail activation. Interestingly, a second regulator of EMT, Twist, was unable to induce EMT or AR expression in prostate cancer cells using similar methodology (Supplementary Figure S3D, S3E). Together with the clinical analyses, these results specifically pinpoint a novel role of Snail in regulating AR expression and localization in prostate cancer.

Given that AR expression and nuclear localization were increased upon Snail activation, we measured the impact of Snail activation on enzalutamide resistance. Compared to vehicle-treated cells, Snail activation with 4OHT in LNCaP95 cells significantly (p<0.001) increased resistance to enzalutamide treatment (Figure 5D) at concentrations physiologically relevant to patients treated with FDA approved doses of enzalutamide [41].

**Figure 6:**
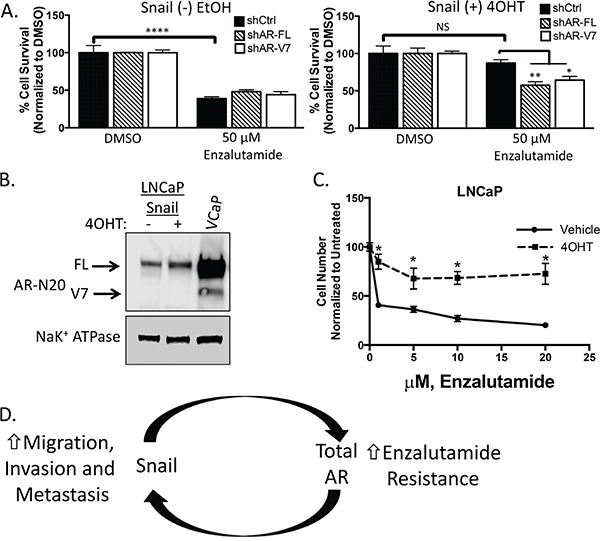
Snail-mediated enzalutamide resistance remains AR dependent **A.** LNCaP95 control or Snail activated cells transduced with the indicated shRNAs were cultured in RPMI 10% CS-FBS containing DMSO or 50 μM enzalutamide. Cells were fixed, permeabilized and stained with Hoechst. Percent cell viability was calculated using ImageJ software analysis of total cell area, *p<0.05, ** p<0.005, ***p<0.0001; one-way ANOVA. **B.** Western blot analysis of AR expression levels in LNCaP cells with or without Snail activation. VCaP is a positive control for AR-V7 detection. **C.** Tamoxifen treated, LNCaP Snail(+) cells are more resistant to enzalutamide inhibited growth. Cell numbers were counted after 7 days in the presence of increasing concentrations of enzalutamide. Graph is representative of two experiments performed in triplicate and are presented as means and SEM, * p<0.05; student's t-test. **D.** A schematic representation of Snail and AR's role in driving metastatic and castrate-resistant disease. Snail mediates metastasis and treatment resistance through the regulation of genes involved in epithelial plasticity and AR, respectively. Likewise, both AR and AR-V7 have been implicated in stimulating epithelial plasticity programs. Thus, one model of disease progression is a feedback loop that can be activated at either Snail or AR with the same outcome, highly metastatic, treatment resistant disease.

To determine whether Snail-mediated enzalutamide resistance was due to upregulation of AR expression and nuclear localization, we used shRNA to stably knockdown either AR-FL or AR-V7 in LNCaP95 cells with inducible Snail (Supplementary Figure S7A). In the presence of each shRNA, vehicle-treated control cells were sensitive to enzalutamide treatment. Similar to above, enzalutamide did not significantly reduce cell growth in 4OHT-treated Snail activated cells expressing a control shRNA. However, depleting either AR-FL or AR-V7 rescued enzalutamide sensitivity in the presence of activated Snail (Figure 6A). These data indicate that both AR-FL and AR-V7 expression are important for Snail-mediated enzalutamide resistance.

Our results suggest that both AR-FL and AR-V7 are important in promoting Snail-mediated enzalutamide resistance. We hypothesized that Snail may require AR-V7 to facilitate enzalutamide resistance. Therefore, we tested the ability of Snail to induce enzalutamide resistance in the absence of AR-V7. The original LNCaP cell line does not have detectable levels of AR-V7 and expression of inducible Snail in LNCaP cells was not sufficient to induce AR-V7 expression (Figure 6B). Conversely, Snail activation increased AR-FL (1.4 fold) expression and nuclear localization (Figure 6B, Supplementary Figure S7B). Importantly, Snail induction led to an increase in enzalutamide resistance (Figure 6C). Together our clinical and *in vitro* data indicate that Snail does not require AR-V7 to mediate enzalutamide resistance. We hypothesize that AR-V7 is important for enzalutamide resistance only if it was previously selected upon during castrate conditions.

Our results suggest that enhanced expression of Snail is important not only in the context of metastatic disease, but also in the setting of human CRPC by contributing to AR expression in response to AR-targeted therapies and thus resistance to AR blockade. We identify a novel function of Snail as a key factor in mediating enzalutamide resistance through the regulation of AR expression and cellular localization to the nucleus. Indeed, depletion of Snail reduces AR-FL and AR-V7 expression and restores enzalutamide sensitivity. These results highlight the potential clinical utility in co-targeting the mechanisms that contribute to the expression and activity of AR-FL and AR-V7 to increase the efficacy of currently used AR-targeted therapies. We also demonstrate that Snail is clinically associated with metastases as compared to localized prostate cancer, and Snail activation is sufficient to enhance the aggressiveness of prostate cancer cells *in vitro*.

## DISCUSSION

### Snail is a novel regulator of enzalutamide resistance

Snail is a transcription factor involved in the regulation of epithelial plasticity through controlling expression of genes involved in migration and invasion. These genes contain E-box elements required for Snail recognition and transcriptional regulation. One hypothesis is that Snail can regulate AR-FL and AR-V7 transcription through the recognition of two E-boxes located near the *AR* transcriptional start site. Interestingly, increased AR transcription rates are directly linked with AR-V7 generation [7]. Alternatively, Snail may indirectly regulate *AR* transcription. A previous study reported that Zeb1, a known Snail target gene, could recognize the E-boxes in the *AR* promoter and drive *AR* transcription [42]. However, we did not observe any effect on AR expression upon knockdown of Zeb1 (Supplementary Figure S7C). In addition, we found that other plasticity regulators, such as TWIST, were not involved in inducing an EMT or in promoting AR expression or enzalutamide resistance, suggesting the specificity of Snail in mediating invasion as well as resistance to AR inhibition. Snail activity and localization can be regulated by various growth factors and kinases, any of which might be targeted to inhibit Snail-mediated enzalutamide resistance and/or metastasis. For example, Snail induction has been linked to nuclear factor-κB (NF-κB) signaling [43], and the NF-κB pathway has also been implicated in enzalutamide resistance and metastatic prostate cancer [44, 45].

In clinical samples, we show that both Snail and AR-V7 expression are increased in metastatic biopsies as compared to primary prostate cancer biopsies, with Snail upregulation observed even in the non-castration resistant metastatic setting. Prior work has detected low levels of AR-variants in localized prostate cancer using RNA-sequencing methods [46]. Our clinical data suggests that there is an increase in AR-V7 expression from localized prostate cancer to metastatic hormone sensitive (HSPC) to CRPC, particularly with enzalutamide/abiraterone resistance. This data is supported by recent clinical findings linking circulating tumor cell AR-V7 expression to primary resistance to both enzalutamide and abiraterone acetate in men with mCRPC [19, 20]. Furthermore, in our *in vitro* model of enzalutamide resistant CRPC, inhibition of Snail decreased AR-FL and AR-V7 expression. Together these results from clinical samples and cell-based models, suggest that Snail is important for early invasion and metastasis in hormone-sensitive disease. However, during further disease progression to CRPC, Snail additionally functions in regulating expression of AR, thereby becoming important for induction of treatment resistance to AR-targeted therapies such as enzalutamide.

### Heterogeneity in our models and patients

Recent studies have highlighted the heterogeneity between and within each prostate cancer patient [46, 47]. In most cases, AR is a clinically relevant therapeutic target; however, the selection pressures imposed by androgen-targeted therapies promotes the emergence of more heterogeneous cell populations. For example, multiple studies have shown that AR inhibition through ADT can promote EMT. Conversely, aberrant AR signaling has been indicated in driving EMT in CRPC (reviewed in [48]). Similarly to these studies that highlight the cell-context-dependent regulation of EMT by AR, a previous study reported that Snail overexpression induced neuroendocrine prostate cancer (NEPC) differentiation in prostate cancer cells [49]. In our study, we did not observe changes in markers of NEPC with Snail activation (data not shown). Acquired random or selected stochastic mutational or epigenetic events can develop during long term serial passaging or be selected upon under castration condition. In the present work, most of the work is modeled as a disease that has already progressed to CRPC, with the next line of therapy being enzalutamide. Conversely, Snail-induced NEPC differentiation was shown in the setting of a hormone-sensitive model of prostate cancer [49].

Another model-dependent effect is that of the relationship between AR-FL, AR-V7, and enzalutamide resistance. Yamamoto et al. (2015) observed that AR-FL drove enzalutamide resistance in LNCaPs harboring the F876L agonistic mutation, while AR-V7 mediated enzalutamide resistance in the 22Rv1 model, in which AR-V7 is expressed at higher levels than full-length AR [50]. These studies highlight the cell context-dependent phenomenon we observe in our model systems. This variability should not be a surprise as the same heterogeneity is observed in patients in the context of genomic events, and different exposures and treatments which may impact gene expression and regulation [46].

### Impeding the inevitable progression to aggression and resistance

The current work identifies Snail, a driver of epithelial plasticity, as a mediator of AR expression and enzalutamide resistance. Conversely, both AR-FL and AR-V7 are also capable of inducing genes associated with epithelial plasticity, including Snail [21, 51–54]. Given the apparent induction of Snail by AR and *vice versa*, one could envision a scenario in which either of these factors initiates gene changes that feed into the other, thereby leading to an aggressive, treatment-resistant tumor. In this scenario, the result would be the same whether the feedback loop started with AR induction or activation of Snail (Figure 6D). Thus, co-inhibition of Snail and AR together may prevent or delay resistance to AR-targeted therapies alone, and several approaches are being developed pre-clinically to inhibit Snail activity [55, 56].

## MATERIALS AND METHODS

### Patient samples

For localized prostate cancer samples (n=188), a tissue microarray (TMA) on a random subset (n=188) of patients in the Shared Equal Access Research Center (SEARCH) database treated at the Durham VA was developed as previously reported [40].

For metastatic prostate cancer samples (n=31) and a second cohort of localized prostate cancer samples (n=29), formalin fixed metastatic tissue was collected from the Duke University pathology department and Duke Cancer Institute Biorepository Core under a separate Duke IRB approved protocol. Clinical data on prior therapy and metastatic site were collected.

### Cell lines

LNCaP95 cells were kindly provided by Dr. Scott Dehm (University of Minnesota) and are reported in previous studies [7]. LNCaP95 are an androgen-independent cell line derived from the parental LNCaP. Both LNCaP and LNCaP95 express mutant AR (T877A). Cells were cultured in RPMI containing 10% charcoal stripped Fetal Bovine Serum (Sigma) and 1% penicillin/streptomycin (Life Technologies). Resistant cell populations were cultured with the addition of 50 μM enzalutamide (provided by Medivation/Astellas). LNCaP95 cells resistant to enzalutamide (LNCaP95-EnzaR) were generated by chronic culture in enzalutamide to a concentration of 50 μM. Cells were authenticated and re-authenticated following enzalutamide resistant through sequencing including the presence of known LNCaP AR ligand binding domain mutation T877A. LNCaP95 cells stably expressing inducible Snail (Addgene plasmid #18798) or indicated shRNA targets were generated by transduction of LNCaP95 cells as described previously [57]. pWZL Blast Snail ER was a gift from Bob Weinberg (Addgene plasmid # 18798) [57]. LNCaP cells were obtained from the Duke Cell Culture Facility and were cultured in RPMI containing 10% Fetal Bovine Serum (Sigma) and 1% penicillin/streptomycin (Life Technologies).

### Cell growth assays

LNCaP95 control and LNCaP95-Enza-R cells were cultured in media containing DMSO (Sigma) or 50 μM enzalutamide for at least one week. Cells were counted using the Countess II (Life Technologies). Average cell number/mL and standard error of the mean (SEM) were calculated from triplicate wells. For LNCaP95 control an Enza-R cells stably expressing shRNAs, cells were treated with DMSO or 50 μM of enzalutamide and counted as above. For LNCaP95 Snail expressing cells, ethanol (EtOH) or 4OHT pre-treated LNCaP95 cells were treated with DMSO or 6.25, 12.5, 25 and 50 μM of enzalutamide. Cell viability was measured after 10 days of drug treatment using the WST-1 assay reagent (Roche). For LNCaP95 Snail cells stably expressing shRNAs, cells were treated with DMSO or 50 μM of enzalutamide for one week. Cells were then fixed in 4% paraformaldehyde (PFA, Sigma) and stained with Hoechst dye (Sigma). Wells were then imaged on an inverted Olympus IX 71 epifluorescence microscope. Total area was calculated from three representative images per well using ImageJ (Version 2.0.0-rc-41/1.50d).

### Real-time quantitative RT-PCR

Total RNA was isolated using the Quick-RNA Miniprep kit (Zymo Research). Total RNA was reverse transcribed using the High-Capacity cDNA Reverse Transcription Kit (Life Technologies). Aliquots of 5-fold diluted reverse transcription reactions were subjected to quantitative (q)PCR with KAPA SYBR FAST master mix using the Vii7 real time-PCR detection system (Applied Biosystems). GAPDH mRNA levels were measured for normalization, and the data are presented as “Relative Expression”. A complete list of primer sequences is provided in the Supplementary Text.

### Immunoblot analyses

For immunoblot analysis cell extracts were mixed with SDS sample buffer and submitted to SDS-PAGE. Following electrophoretic transfer onto nitrocellulose, the filters were blocked, incubated with antibodies and developed using the Odyssey-FC imager (LI-COR).

For nuclear and cytoplasm fractionation, nuclear and cytoplasmic extraction was performed according to the manufacture's protocol using the NE-PER kit (ThermoScientific). A complete list of primary antibodies and their dilutions is provided in the Supplementary Text. All immunoblot images are of cropped blots to improve the clarity and conciseness of the manuscript and retain all important bands.

### Immunofluorescence staining

For immunofluorescence (IF), cells were fixed in 4% PFA, permeabilized with 0.2% Triton X-100, and stained with Hoechst. Cells were blocked with 5% bovine serum albumin (BSA, Sigma) prior to incubation with primary antibodies. Cells were incubated in Alexa Fluor secondary antibodies (Life Technologies) and then imaged on an inverted Olympus IX 71 epifluorescence microscope or using Cellomics Software for nuclear to cytoplasmic quantification.

### Migration and invasion assays

Culture medium was added to the lower wells of Boyden chambers (BD Bioscience), and cells were added to the upper chambers. Migratory cells attached to the lower side of the inserts were fixed with 4% PFA, permeabilized with 0.2% Triton X-100, and stained with Phalloidin (Life Technologies). The inserts were washed with 1× PBS (Life Technologies) and imaged using a 4× objective on an inverted Olympus IX 71 epifluorescence microscope and analyzed with ImageJ. Matrigel invasion assays were performed using Matrigel-coated 24-well transwell plates (BD Biosciences) as described above.

### Immunohistochemistry

We performed antibody optimization and analytic validation for all antibodies as previously described [40]. An expert prostate cancer pathologist blinded to outcomes evaluated antibody staining in parallel with hematoxylin and eosin. Scoring of each biomarker used a 0 to 3 scale for both intensity and a <25%, <50%, <75%, <100% scale for frequency of expression in each tumor sample.

### RNA-ISH

For AR-V7 RNA-ISH, formalin fixed paraffin-embedded metastatic tumor slides were incubated with the AR-V7 probe (401221) using the RNAscope 2.0 HD Brown Assay from Advanced Cell Diagnostics according to the manufacturer's protocol. An expert pathologist scored AR-V7 mRNA expression using a 0-4 scale following the guidelines outlined in the manufacture's protocol.

### Statistical analysis

Data are shown as means ± SEM. Student's t-test or multiple group comparison was performed by one-way ANOVA followed by the Sidak method for comparison of means. P≤0.05 is considered significant. Differences in Snail and AR-V7 expression between localized and metastatic samples were analyzed using a Chi-square test. Data available through the TCGA was analyzed using the Kruskal Wallis test using JMP (version pro 12). All other analyses were performed using Prism (version 6.0d).

## SUPPLEMENTARY MATERIALS FIGURES


