# Supplementary material for: Snail promotes resistance to enzalutamide through regulation of androgen receptor activity in prostate cancer
Source: Oncotarget. 2016 Jul 7;7(31):50507–21. doi: 10.18632/oncotarget.10476 (PMC5226599; doi:10.18632/oncotarget.10476)
Supplement: Supplementary file 1 [file oncotarget-07-50507-s001.pdf]

Snail promotes resistance to enzalutamide through regulation of androgen receptor activity in prostate cancer

SUPPLEMENTARY MATERIALS AND METHODS

TCGA analysis

The results published here are in part based upon data generated by The Cancer Genome Atlas (TCGA) Research Network: http://cancergenome.nih.gov. Data available from TCGA was analyzed using the Kruskal Wallis test to evaluate the correlation between Snail expression and Gleason score from prostate cancer patients. Boxplots were created using RStudio (version 0.98.1091).

Analysis of F876L status

The ligand binding domain (LBD) of AR was sequenced from cDNA generated from LNCaP95 EnzaR cells using forward primer 5' ATTGCGAGAGAGCTGCATCA 3' and reverse primer 5' TTCTCGTCACTATTGGCCTC 3' and analyzed using Sanger sequencing.

Real-time quantitative RT-PCR

Total RNA was isolated using the Quick-RNA Miniprep kit from Zymo Research. Total RNA (1 µg) was reverse transcribed in a volume of 20 µl using the High-Capacity cDNA Reverse Transcription Kit (Life Technologies). Aliquots (2µl) of 5-fold diluted reverse transcription reactions were subjected to quantitative (q)PCR in 10-20µl reactions with KAP SYBR FAST Universal 2x qPCR Mastermix and primers below using a Vii7 real time-PCR detection system (Applied Biosystems). GAPDH mRNA levels were measured by qPCR in replicate samples as a housekeeper gene for normalization and the data are presented as "Relative Expression".

Immunohistochemistry

Twenty-nine prostatectomies from the Duke University Biospecimen Repository and Processing Core were stained for AR or Snail expression. An expert prostate cancer pathologist blinded to outcomes evaluated antibodies against AR and Snail in parallel with hematoxylin and eosin. Scoring is based on frequency of expression of each biomarker in each tumor sample.

Immunoblot analyses and immunofluorescence

Whole cell lysates were analyzed by SDS-PAGE and electrophoretic transfer as described in the main text with the following antibodies and concentrations. For immunofluorescence (IF), cells were fixed in 4% PFA, permeabilized with 0.2% Triton X-100, and stained with Hoechst. Cells were blocked with 5% bovine serum albumin (BSA, Sigma) prior to incubation with primary antibodies. Cells were incubated in Alexa Fluor secondary antibodies (Life Technologies) and then imaged on an inverted Olympus IX 71 epifluorescence microscope. ImageJ (Version 2.0.0-rc-41/1.50d) was used to quantify nuclear localization of Snail.

shRNA target sequences

Target	Target Sequence	Company
AR-FL-15	ATGTGTGACTTGATTAGCAGGT	Sigma
AR-FL-18	CACCAATGTCAACTCCAGGAT	Sigma
AR-V7-1	GTAATAGTGGTTACCACTC	Cloned
AR-V7-2	AGGCTAATGAGGTTTATTT	Cloned
Snail-19	CCAGGCTCGAAAGGCCTTCAACT	Sigma
Snail-22	GCAGGACTCTAATCCAGAGTT	Sigma

Primer	Sequence (5’ to 3’)
AR-FL -F QPCR	GCCTTGCTCTCTAGCCTCAA
AR-FL-R QPCR	GGTCGTCCACGTGTAAGTTG
AR-V7-F QPCR	CCATCTTGTCGTCTTCGGAAATGTTATGAA
AR-V7-R QPCR	TTTGAATGAGGCAAGTCAGCCTTTCT
GAPDH-F	AGCCACATCGCTCAGACAC
GAPDH-R	GCCCAATACGACCAAATCC
CDH1 -F	GGTCTGTCATGGAAGGTGCT
CDH1-R	GATGGCGGCATTGTAGGT
SNAIL F1	CCCCAATCGGAAGCCTAACT
SNAIL R1	AGGATCTCCGGAGGTGGGAT
ZEB1-F	GCATACAGAACCCAACTTGAACGTC
ZEB1-R	CGATTACACCCAGACTGCGTC
VIMENTIN-F	CTTCGCCAACTACATCGACA
VIMENTIN-R	CGCATCTCCTCCTCGTAGA
TWIST1-F	CATGGCTAACGTGCGGGA
TWIST1-R	CGCCAGTTTGAGGGTCTGAA
JUP-F	GACAGAAAAGTGCCTGAGCTG
JUP-R	CTATCCCAAGAAAGACCCTACG
O-CADHERIN-F	CACCGTACAGTTGGTGGAAG
O-CADHERIN-R	CCTTCATTTTTGGTTACGTGGT
ESRP1-F	TTTTGAATCCACGAGCACTG
ESRP1-R	GCCAAGACTATTAGGCGAACC
CDH2-F	CTCCATGTGCCGGATAGC
CDH2-R	CGATTTCACCAGAAGCCTCTAC
GRHL2-F	CATGTCACAAGAGTCGGACAA
GFHL2-R	GGGCACTAAGGCCACTAGTCT
SLUG-F	TGGTTGCTTCAAGGACACAT
SLUG-R	GCAAATGCTCTGTTGCAGTG
ZEB2-F	TCGGCTGCTTCATTGATAAGAGC
ZEB2-R	GCTTCATATTGCTGAGGATGACGGT

Antibody	Catalog #	Company	Dilution
AR (C19)	sc-815	Santa Cruz Biotechnologies	1:100 IF
AR (N20)	sc-816	Santa Cruz Biotechnologies	1:100 IF 1:1000 W
Snail (C15D3)	3879S	Cell Signaling	1:200 IF 1:2000 W
Vimentin	MCA862	BioRad	1:500 W
Ecadherin	610182	BD Transduction Laboratories	1:10000 W
hnRNPA1 (9H10)	ab5832	AbCam	1:1000 W
ZEB1 (H-102)	sc-25388	Santa Cruz Biotechnologies	1:500 IF
alpha-tubulin	322500	Invitrogen	1:1000 W
PTB	NA	[1]	1:4000 W
Snail	Ab85936	Abcam	1:500 IHC
Pan-Cytokeratin	MCA1907T	AbD Serotec	1:100 IHC
Vimentin	M7020	Dako	1:150 IHC

REFERENCES

1. Wagner EJ, Carstens RP, Garcia-Blanco MA. A novel isoform ratio switch of the polypyrimidine tract binding protein. Electrophoresis. 1999; 20:1082-6.

SUPPLEMENTARY FIGURES

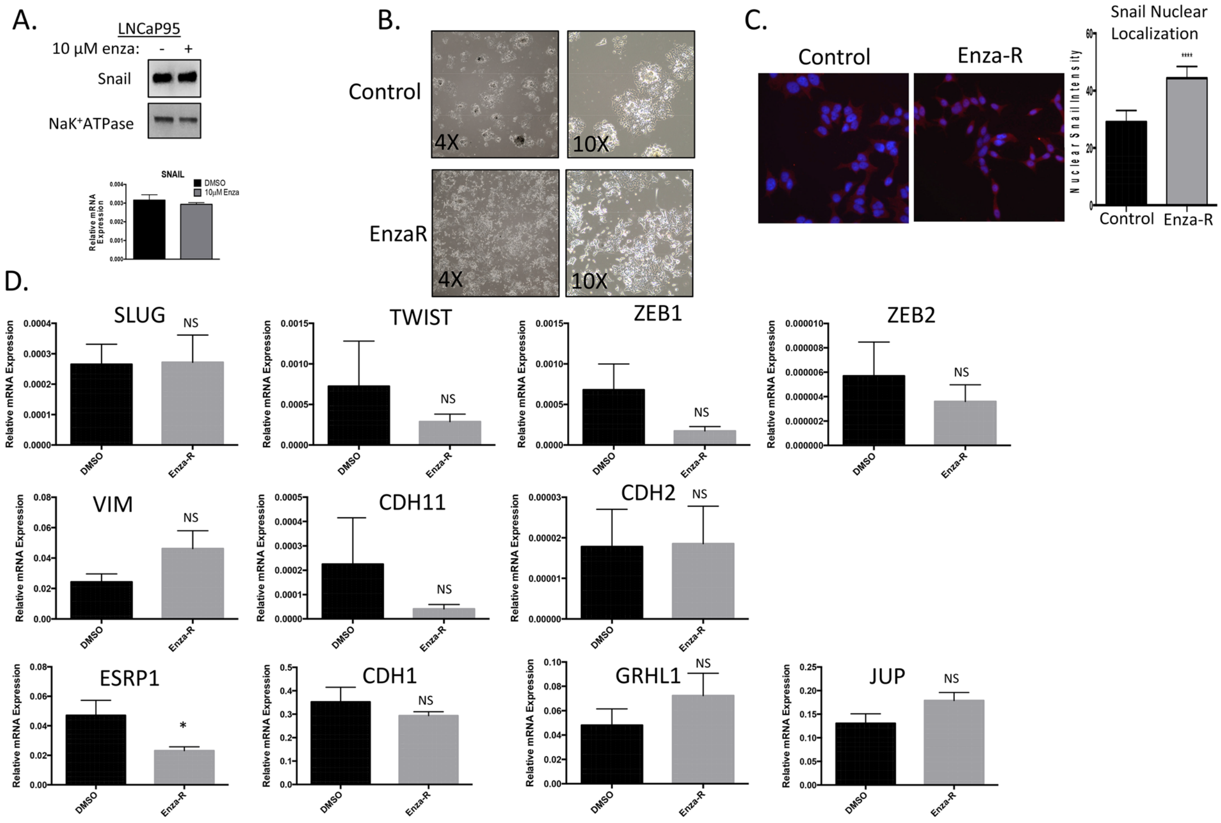

Supplementary Figure S1: A. Protein and mRNA expression in parental LNCaP95 cells treated acutely (6 days) with 10μM enzalutamide. **B.** Images of control and EnzaR cells at 4X and 10X magnification. **C.** Representative images and quantification for Snail nuclear localization in control and EnzaR LNCaP95 cells. Blue: Hoechst stained nuclei; Red: Snail. **D.** Relative mRNA levels of Slug, Twist1, Zeb1, Zeb2, Vim, CDH11, CDH2, CDH1, ESRP1, GRHL1 and JUP were measured using qPCR and normalized to GAPDH.

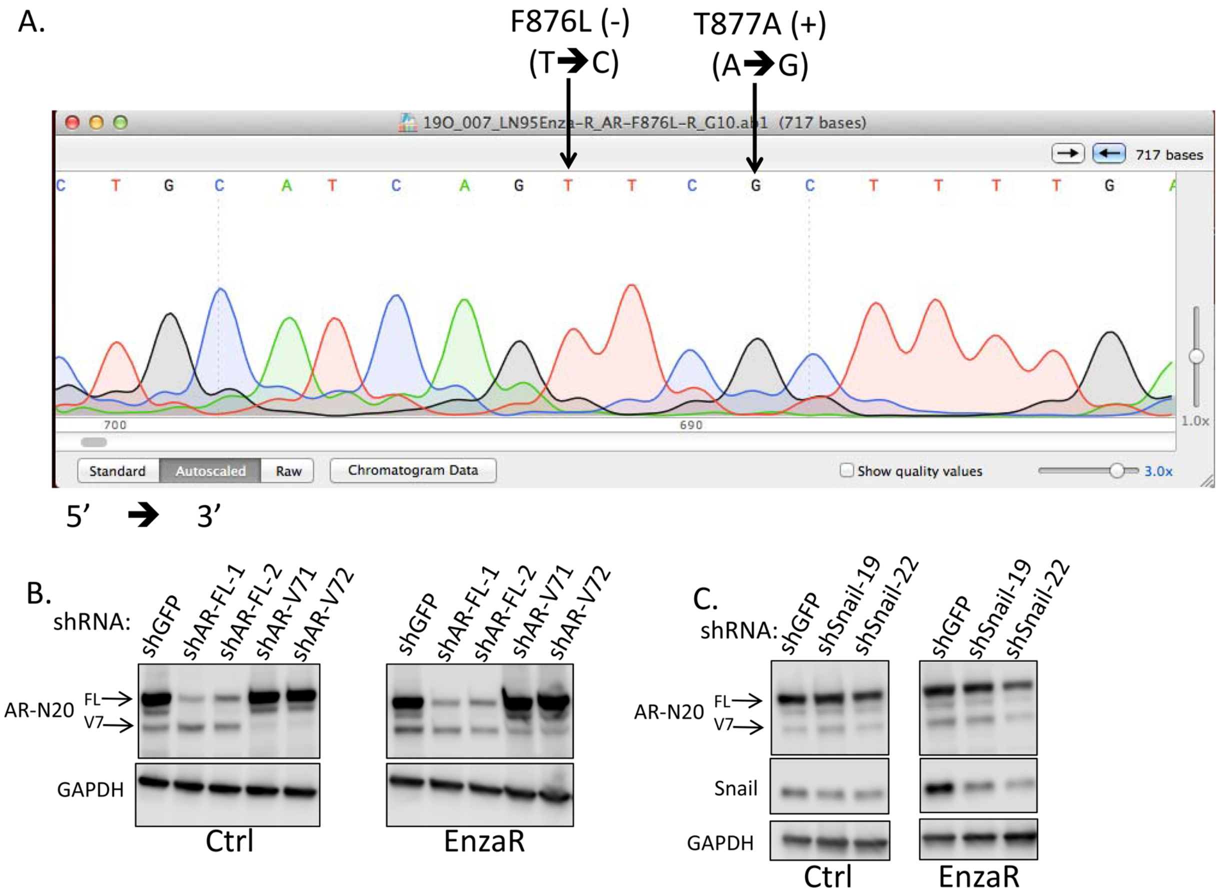

Supplementary Figure S2: A. Sanger sequencing trace plots of mRNA isolated form LNCaP95-EnzaR cells. Arrows indicate the absence of the agonistic mutation for enzalutamide (F876L), but retention of the T877A mutation from parental LNCaP cells. **B-C.** Western analysis of AR (B) or Snail (C) knockdown in LNCaP95 cells.

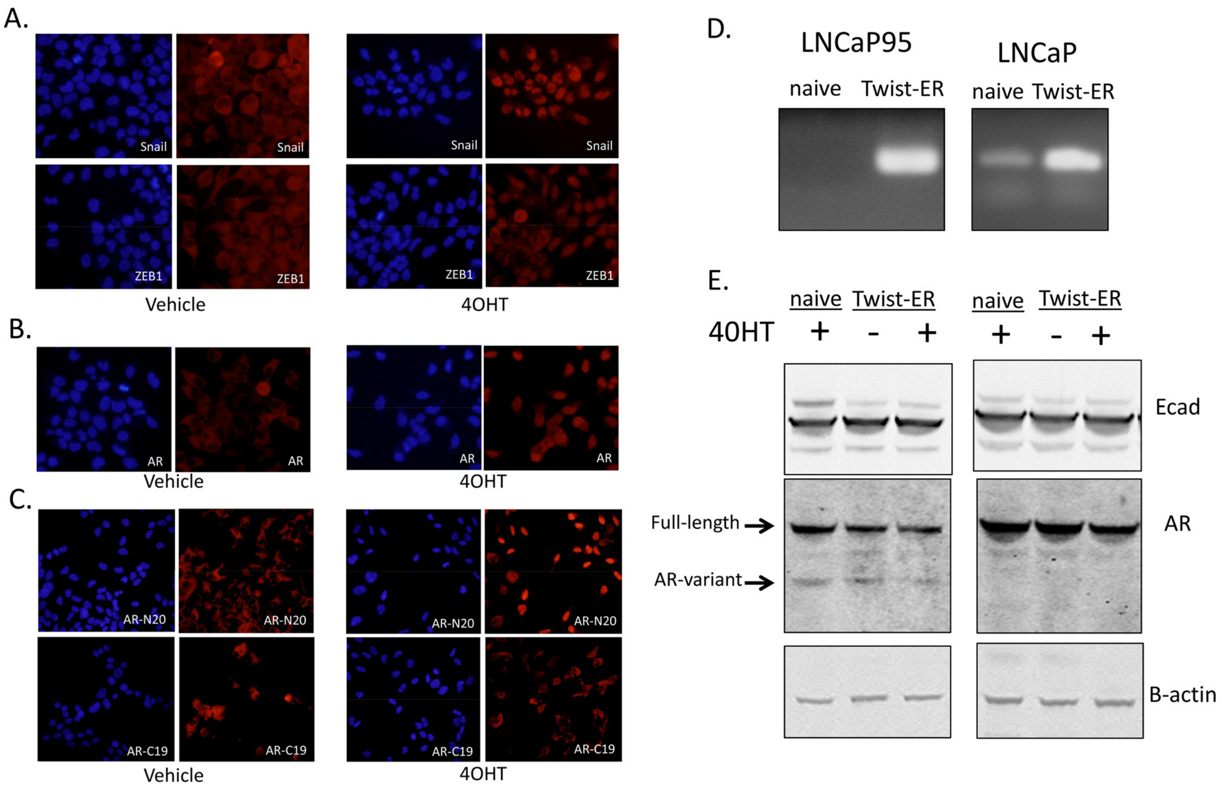

Supplementary Figure S3: A-C. The original single-channel images used to make the merged file shown in the main text. **D.** Twist mRNA levels in LNCaP95 or LNCaP cells transduced with inducible Twist-ER plasmid. **E.** Twist activity was induced with 4OHT and the impact on E-cadherin, AR or AR-V7 expression was measured by western blot in LNCaP95 and LNCaP cells.

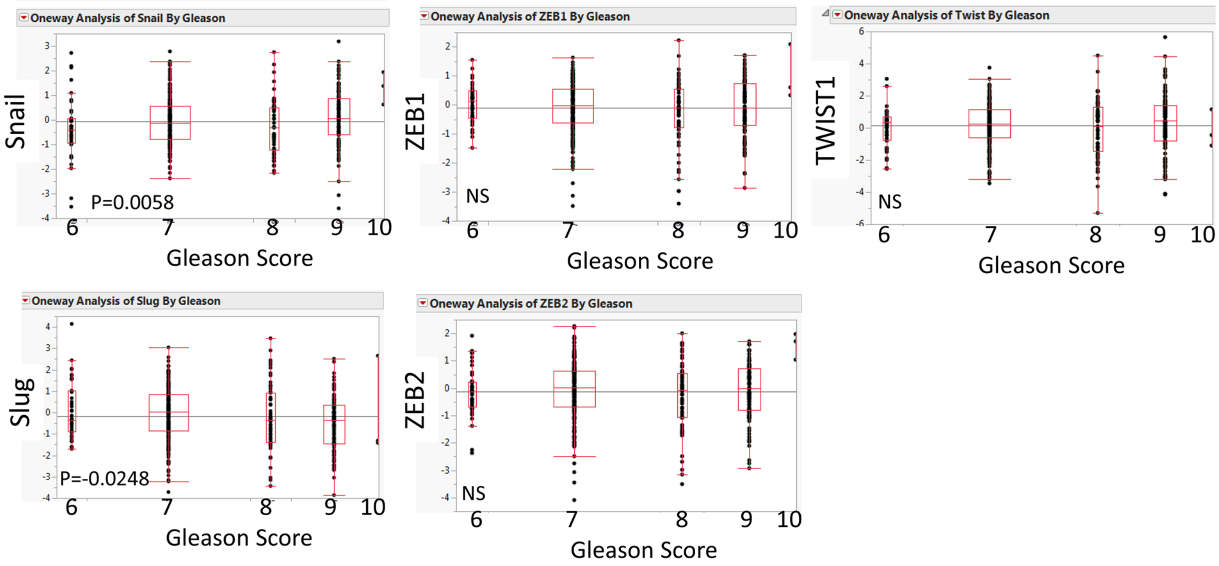

Supplementary Figure S4: TCGA analysis of Snail, Slug, ZEB1, ZEB2 and TWIST1 expression with Gleason score from prostate cancer patient samples.

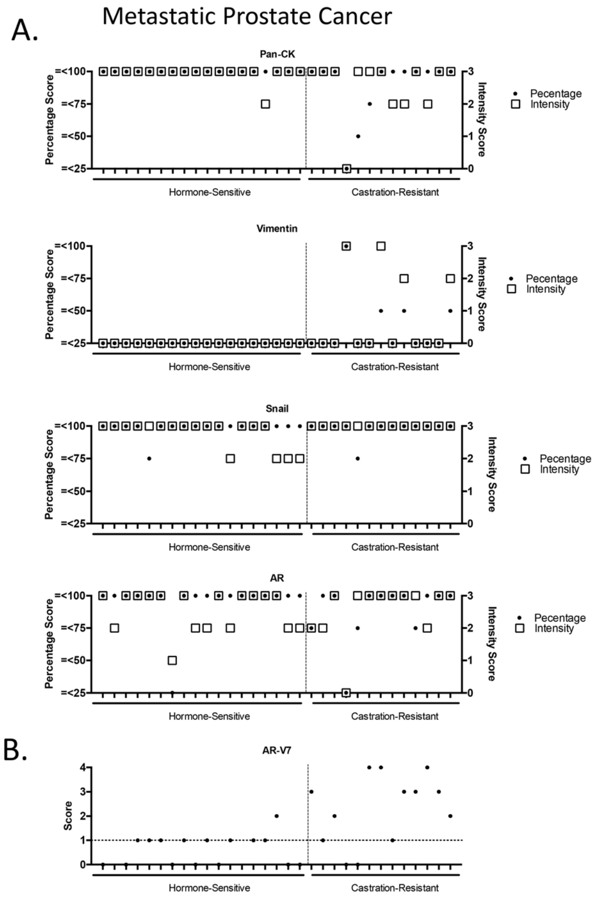

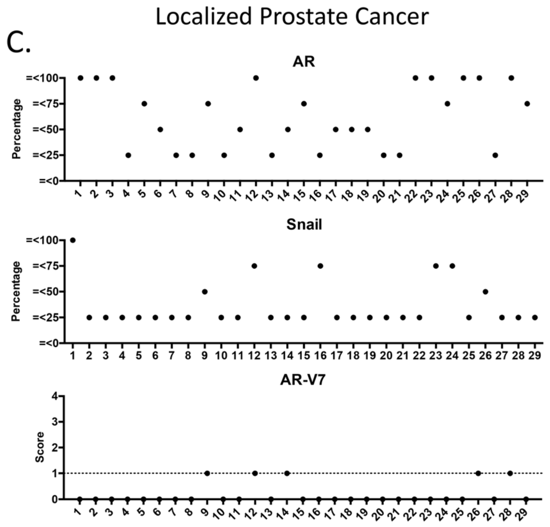

D.

Clinical and Pathologic Characteristics of all Patients	N	%	AR-V7 Positive Patients N	AR-V7 Positive Patients %
**Total # patients**	31	100.0	10	32.3
**Site of Biopsy**				
Brain	3	9.7	3	100.0
Bone	4	12.9	1	25.0
Visceral	5	16.1	1	20.0
Lymph Nodes	22	71.0	4	18.2
Other	1	3.2	1	100.0
**Castration Resistance**				
No	18	58.1	1	5.5
Yes	13	41.9	9	69.2
**Prior Abiraterone or Enzalutamide**				
No	25	80.6	5	20.0
Unknown	2	6.5	1	50.0
Yes	4	12.9	4	100.0

Supplementary Figure S5: IHC and RNAish analysis of metastatic (A and B) and localized (C) biopsies from prostate cancer patients. A. Each sample was given two scores based on percentage of positive cells (closed dot) or intensity of staining (open box). Hormone-sensitive and castrate-resistant patients are separated by a dashed vertical line. **B.** Analysis of RNAish for AR-V7 in metastatic biopsies. Each sample was given a score (0-4) based on percentage of positive cells and intensity of staining. Background staining is indicated with a horizontal dash line as determined using AR-V7 negative (PC3) and positive (LNCaP95, 22Rv1) control cell lines. **C.** IHC or RNAish analysis of 29 localized prostate tumors. The percentage of positive cells was used to score protein expression of AR or Snail. For RNAish, a 0-4 scale as in panel B. **D.** Clinical and pathologic characteristics of all patients.

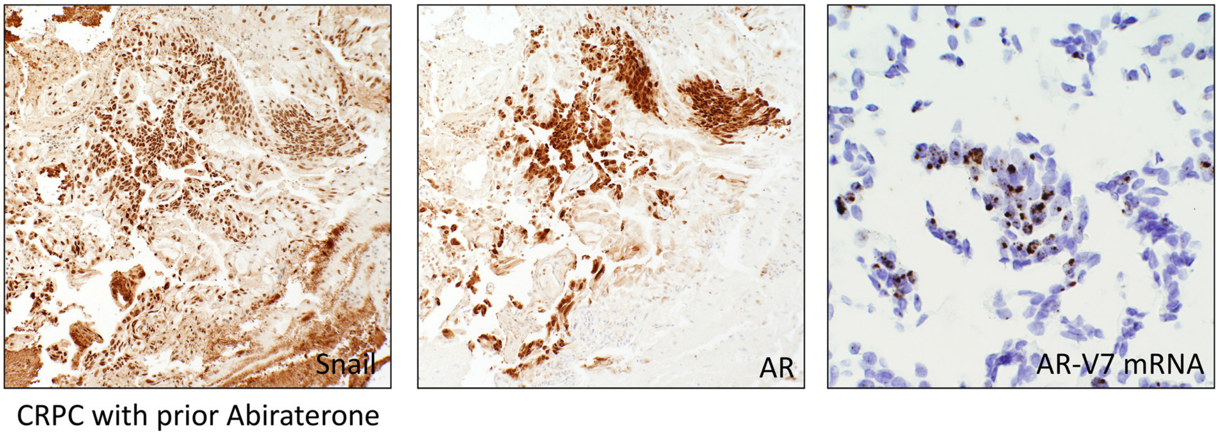

Supplementary Figure S6: Images from a brain metastases stained for Snail and AR by IHC and AR-V7 by RNAish.

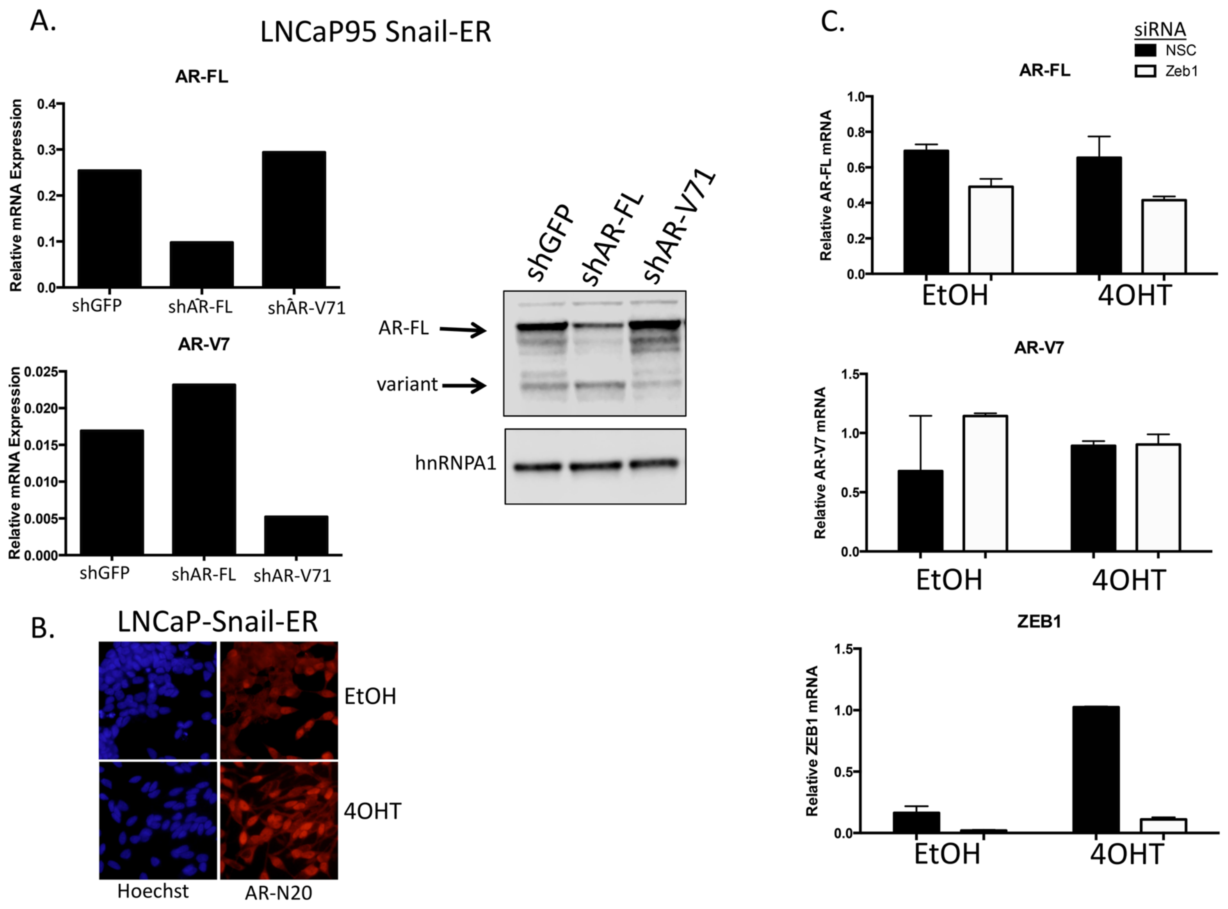

Supplementary Figure S7: A. qPCR and western analysis of AR-FL or AR-V7 knockdown in LNCaP95 cells transduced with inducible Snail. **B.** Representative images from immunofluorescence staining in LNCaP cells expressing Snail-ER for total AR (Blue: Hoechst stained nuclei; Red: AR) **C.** qPCR analysis of AR-FL, AR-V7 and Zeb1 mRNA expression in cells transfected with control or Zeb1 siRNAs.
